# Carriers of Parkinson’s disease-linked SNCA Rep1 variant have greater non-motor decline: a 4 year follow up study

**DOI:** 10.18632/aging.206196

**Published:** 2025-02-03

**Authors:** Aarthi Santhanakrishnan, Yi Jayne Tan, Seyed Ehsan Saffari, Yi Zhao, Ebonne Y.L. Ng, Samuel Y.E. Ng, Nicole S.Y. Chia, Xinyi Choi, Dede Heng, Shermyn Neo, Zheyu Xu, Kay Yaw Tay, Wing Lok Au, Eng-King Tan, Louis C.S. Tan, Adeline S.L. Ng

**Affiliations:** 1 Lee Kong Chian School of Medicine, Nanyang Technological University, Singapore; 2 Department of Neurology, National Neuroscience Institute, Tan Tock Seng Hospital, Singapore; 3 Center for Quantitative Medicine, Duke-NUS Medical School, National University of Singapore, Singapore; 4 Department of Clinical Translational Research, Singapore General Hospital, Singapore; 5 Department of Neurology, National Neuroscience Institute, Singapore General Hospital, Singapore; 6 Neuroscience and Behavioural Disorders, Duke-NUS Medical School, Singapore

**Keywords:** genetics, Parkinson’s disease, cognition, memory, apathy

## Abstract

Alpha-synuclein gene promoter (SNCA Rep1) polymorphism has been linked to Parkinson's Disease (PD) susceptibility and motor symptom severity, but less is known about its longitudinal relationship with non-motor symptom severity. To address this gap, this is the first longitudinal study over 4 years investigating the relationship between Rep1 allele length and non-motor function amongst 208 early PD patients grouped into long (*n* = 111) vs. short (*n* = 97) Rep1 allele carriers. Long Rep1 carriers demonstrated faster decline in global cognition (*p* = 0.023) and increasing apathy (*p* = 0.027), with greater decline in attention and memory domains (*p* = 0.001), highlighting the utility of Rep1 polymorphism in stratifying patients at risk of non-motor symptom decline.

## INTRODUCTION

Parkinson’s disease (PD) is characterized by both motor and non-motor symptoms, including depression, anxiety, and global cognitive dysfunction. Neuropathological hallmarks include degeneration of dopaminergic neurons in the substantia nigra leading to motor symptoms and α-synuclein containing Lewy bodies in cortical and limbic areas leading to cognitive and neuropsychiatric symptoms [1, 2]. Rep1 is a polymorphic dinucleotide repeat sequence located 10kb upstream of the α-synuclein gene (*SNCA)* gene site in its promoter region. Associations of Rep1 allele length with PD suggests longer allele length being more common amongst PD patients [3, 4], associated with earlier PD onset [5] and faster motor symptom progression [6]. While a recent study investigated longitudinal association of Rep1 allele length with severity of specific symptoms in PD [7], the scope was restricted to analysing the presence or absence of visual hallucinations or dementia. Hence, to address this gap, we investigated the longitudinal relationship between Rep1 allele length and a wider range of non-motor symptoms, including global cognition, in early PD patients over a period of 4 years. We hypothesized that longer *SNCA* Rep1 allele carriers will show greater increase in non-motor impairment, including cognition, compared to shorter allele carriers over 4 years.

## RESULTS

### Baseline characteristics

208 patients were included in this study. Baseline demographics are summarized in Table 1A. There was no significant difference between the short and long Rep1 carrier groups in gender proportion, mean age at diagnosis, disease duration, *APOE4* carrier proportion and baseline motor and cognitive function i.e., H&Y stage and MoCA scores respectively. At baseline, analysis of covariance revealed no significant difference in non-motor symptom outcomes between both groups (Table 1B). There was no significant association between Rep1 length and motor outcomes (measured by MDS-UPDRS part III motor scores (*p* = 0.953) and H&Y stages (*p* = 0.094)), adjusting for age, gender and disease duration.

**Table 1:** Clinical characteristics and non-motor symptom outcomes of PD patients at baseline.

A. Characteristic^*^	**Rep1-Short** **(*n* = 97)**	**Rep1-Long** **(*n* = 111)**	***p* ^a^**
Age, years	63.02 ± 9.92	64.55 ± 8.04	0.222
Sex, male	56 (57.7%)	64 (57.6%)	0.991
Age at onset, years	62.1 ± 9.8	63.4 ± 8.2	0.318
Disease duration, years	1.00 (0.50–1.40)	1.00 (0.70–1.39)	0.719
*APOE4* carriers	24 (24.7%)	20 (18.0%)	0.236
Y1 H&Y stage	1.77 ± 0.475	1.88 ± 0.458	0.054
Y1 MDS-UPDRS part III	21.2 ± 9.65	21.5 ± 10.2	0.837
Y1 MoCA	24.92 ± 3.35	25.36 ± 3.81	0.378
Y1 LEDD	187.5 (100–237.5)	131.5 (0.00–259.375)	0.126
B. Outcome^*^	**Rep1-Short** **(*n* = 97)**	**Rep1-Long** **(*n* = 111)**	***p* ^b^**
MoCA	24.79 ± 3.32	25.32 ± 3.80	0.067
MMSE	26.69 ± 2.76	26.91 ± 2.84	0.218
NMSS	19.02 ± 19.27	20.93 ± 17.89	0.439
PDQ-R	2.64 ± 3.50	2.69 ± 3.42	0.914
PDQ-T	8.25 ± 10.93	8.41 ± 10.68	0.914
HAD-A	2.62 ± 2.71	2.25 ± 2.73	0.570
HAD-D	2.86 ± 2.83	3.18 ± 2.61	0.277
ASSc	9.26 ± 6.12	9.08 ± 6.73	0.842
FSST	27.27 ± 13.26	29.57 ± 13.93	0.198
FSSM	3.03 ± 1.47	3.29 ± 1.55	0.198
ESST	6.15 ± 4.13	6.19 ± 4.07	0.773
PSQI	4.65 ± 3.30	4.91 ± 3.31	0.627
NMSS_D1	0.89 ± 2.06	0.68 ± 1.66	0.643
NMSS_D2	3.21 ± 4.83	3.50 ± 5.14	0.568
NMSS_D3	2.74 ± 7.24	1.64 ± 3.51	0.328
NMSS_D4	0.55 ± 1.70	0.68 ± 3.00	0.703
NMSS_D5	1.50 ± 3.34	2.22 ± 4.15	0.084
NMSS_D6	2.04 ± 4.18	2.52 ± 3.74	0.534
NMSS_D7	5.15 ± 5.80	5.27 ± 7.89	0.905
NMSS_D8	1.10 ± 3.41	1.30 ± 3.27	0.661
NMSS_D9	1.84 ± 2.86	3.10 ± 4.81	0.067

^*^Continuous variables are reported as mean ± standard deviation or median (25th–75th percentile (weighted average)) while categorical variables are reported as frequency (%). ^a^Two-sided two-sample *t*-test for Age and Y1 MoCA, Mann-Whitney test for Disease Duration, Y1 H&Y stage and Y1 LEDD, Chi-squared test for Sex and *APOE4* carriers. *p* < 0.05. PD, Parkinson's Disease; Y1 H&Y stage, Hoehn and Yahr stage at baseline; Y1 MoCA, Montreal Cognitive Assessment score at baseline; Y1 LEDD, Levodopa Equivalent Daily Dose at baseline. ^b^Multivariate analysis of covariance conducted on logarithmic transformed values of right-skewed variables: NMSS, PDQ-R, PDQ-T, HAD-A, HAD-D, ASSc, FSST, FSSM, ESST, PSQI, NMSS_D1-9.* p* < 0.05. Potential confounders controlled for are age, gender, disease duration, *APOE4* status, baseline H&Y status (and baseline MoCA score for evaluation of ASSc and NMSS_D5). Abbreviations: MoCA: Montreal Cognitive Assessment score; MMSE: Mini mental state exam; NMSS: Non-motor Symptoms Scale; PDQ-R: Parkinson’s Disease Questionnaire-8 item Raw score; PDQ-T: Parkinson’s Disease Questionnaire-8 item Total score; HAD-A: Hospital Anxiety and Depression Scale - Anxiety; HAD-D: Hospital Anxiety and Depression Scale - Depression; ASSc: Apathy Scale; FSST: Fatigue Severity Scale Total score; FSSM: Fatigue Severity Scale Mean score; ESST: Epworth Sleepiness Scale; PSQI: Pittsburgh Sleep Quality Index; NMSS_D1–D9: Non-motor Symptoms Scale Domains 1–9.

### Rep1 allele length and non-motor symptoms progression over 4 years

**Figure 1:**
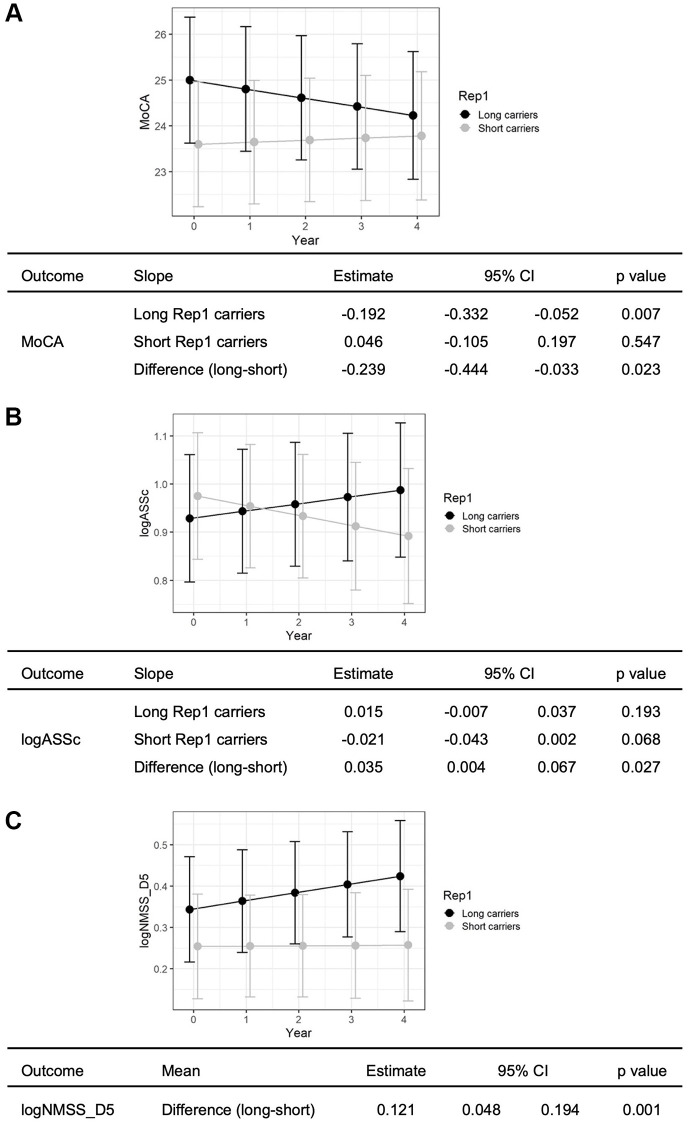
**Long Rep1 allele carriers showed significant decline in non-motor functions.** Specifically significant findings were noted in (**A**) global cognitive function, measured by MoCA, (**B**) apathy, measured by Apathy scale and (**C**) attention and memory, measured by NMSS Domain 5. Results are generated using linear mixed model analysis, controlling for potential confounders including age, gender, disease duration, *APOE4* status, baseline H&Y status (and baseline MoCA score for evaluation of apathy and attention and memory).

Using linear mixed model, we examined the associations between Rep1 length and non-motor outcomes over 4 years in i) global cognition (MMSE, MoCA), ii) non-motor symptoms outcomes (10 tests), and iii) 9 subdomains in NMSS. Long Rep1 carriers showed significant decline in global cognitive function (MoCA) compared to short allele carriers, after adjusting for age, gender, disease duration, H&Y stage and *APOE4* status (β = −0.239, *p* = 0.023) (Figure 1A). Next, Long Rep1 carriers showed significantly more apathy on the Apathy Scale after adjusting for age, gender, disease duration, baseline MoCA score and H&Y stage and *APOE4* status (β = 0.035, *p* = 0.027) (Figure 1B), though this was not significant after correction for multiple testing (0.05/10 tests = 0.005). No significant associations were observed in other non-motor symptom tests (Supplementary Figure 1). Lastly, Long Rep1 carriers had significantly higher scores for NMSS Domain 5, specific for deficits in attention and memory, after adjusting for age, gender, disease duration, baseline MoCA score and H&Y stage and *APOE4* status (β = 0.121, *p* = 0.001) (Figure 1C). After multiple testing correction, positive association between Rep1 length and clinical outcomes remained significant for NMSS Domain 5 scores (0.05/9 domains = 0.005). These results remained after adjusting for the levodopa equivalent daily dose of patients at baseline.

## DISCUSSION

In this prospective 4-year study of well-characterized early PD cases, we demonstrate for the first time that long Rep1 allele length carriers show greater decline in global cognition on the MoCA, greater decline in attention and memory subdomains on the NMSS, and worsening apathy, compared to carriers of the short allele.

The *SNCA* gene encodes for the alpha-synuclein protein which accumulates within neurons as Lewy bodies resulting in neurodegeneration. Longer SNCA Rep1 allele length has been associated with increased risk and earlier onset of PD [5] and worse cognitive outcomes [7]. Pathologically, longer 261bp Rep1 alleles have been associated with higher synuclein levels in murine brain [8] and shorter 259bp alleles with lower levels in human peripheral blood mononuclear cells [9], with the shorter Rep1 allele being associated with significantly lower levels of SNCA in the substantia nigra and temporal cortex [10]. This suggests that longer Rep1 allele length, through greater pathological burden, might be associated with greater disease severity or steeper functional decline. Noncoding simple sequence repeat variants such as Rep1 may also exert pathogenicity by regulating gene expression via the non-consensus transcription factor binding mechanism to affect the binding of transcription factors from the GATA family to its specific site located next to the Rep1 repeat [11]. These results provide biological support for our findings of worse clinical outcomes in long Rep1 allele carriers over 4 years.

Cognitive decline is one of the more common and debilitating non-motor symptoms in PD and post-mortem studies of PD dementia report 10x higher Lewy body counts in the neocortex, limbic cortex and amygdala compared to PD patients without dementia [12]. Deficits in memory and attention are frequently reported in PD patients. Apart from cortical Lewy body neuropathology, reduced dopamine transporter availability causing nigro-caudate dysfunction and dopamine depletion in the anterior putamen has also been associated with reduced performance in memory and attention tasks in PD [13]. Hippocampal SNCA accumulation has been associated with memory impairment in transgenic mice, with suppression of *SNCA* gene expression resulting in partial clearance of pre-existing pathology, reversal of synaptic defects and improved memory [14]. Previous research supports our finding through a cross-sectional study of a similar population, demonstrating cognitive decline as indicated by MMSE scores [15]. In our study, however, we did not observe a significant association between cognitive decline based on the MMSE score with Rep1 allele length, but there was a significant association with allele length and MoCA scores, potentially reflecting the increased sensitivity of the latter in detecting cognitive decline [16].

Apathy remains a common non-motor symptom in early PD and contributes to reduced quality of life and caregiver burden. Loss of dopaminergic and noradrenergic innervation of the ventral striatum and serotonergic lesions within the right-sided anterior caudate nucleus and orbitofrontal cortex has been observed to play a role in apathy in PD [17]. Significantly higher SNCA cerebrospinal fluid oligomer levels have also been reported in PD patients with apathy compared to those without, possibly related to increased oxidative stress induced by SNCA oligomers in the brain [18].

Future research could focus on further detailed analysis of non-motor symptoms which are only briefly screened for under NMSS subdomains. Using specialised scales to assess symptoms such as psychosis and gastrointestinal disturbances rather than broad subdomain scores could enable a more comprehensive analysis of specific symptomology. Overall, this is the first study reporting 4-year longitudinal data of over 200 early-PD participants with Rep1 genotyping and comprehensive clinical and neuropsychological testing. Our findings are important for facilitating early identification and stratification of PD patients at increased risk of cognitive and functional decline and promote earlier implementation of individualized therapeutic strategies for patients.

## MATERIALS AND METHODS

In this prospective study, 208 early PD patients were recruited between 2014 and 2019, and were followed up for 4 years at the National Neuroscience Institute, Singapore, as part of the Parkinson’s Disease Longitudinal Singapore (PALS) study with inclusion criteria as previously described [15]. All participants fulfilled the National Institute of Neurological Disorders and Stroke clinical criteria for PD. Early PD was defined as patients with PD diagnosis made less than a year before recruitment and motor symptoms onset less than two years before diagnosis. Patients with a history of clinical or symptomatic stroke, active malignancy, end-organ failure, significant orthopedic abnormalities which affect movement and/or other significant neurological or psychiatric conditions were excluded. The PD cohort is population-based and is expected to reflect the range of genetic distribution as well as common disease phenotypes more accurately. Ethics approval was obtained from the Singapore Health Services Centralised Institutional Review Board (CIRB, Ref No. (2019/2433) for the use of human participants in this study, and all participants provided informed written consent.

The sample size for the primary study, the PALS, was calculated to achieve adequate power for its primary objectives. Specifically, based on anticipated conversion rates from PD to PD-dementia and from normal cognition to PD-MCI over a 5-year period, a total of 250 early PD patients and 150 control subjects were deemed necessary to achieve a statistical power of 0.8. This calculation also accounted for a 25% attrition rate, leading to a target sample of at least 124 participants in the first year. As this paper reports on a secondary analysis of data collected from the primary study, no new sample size calculation was performed for the current analysis, as it utilizes the data from the already adequately powered primary study.

Global cognition was examined using the Montreal Cognitive Assessment (MoCA) and Mini Mental State Examination (MMSE). Non-motor symptoms were examined using the Non-Motor Symptoms Scale, Parkinson’s Disease Questionnaire-8 item, Hospital Anxiety and Depression Scale, Apathy Scale, Fatigue Severity Scale, Epworth Sleepiness Scale, and Pittsburgh Sleep Quality Index. All patients were on levodopa treatment and examined while in the “ON” state.

Genomic DNA was extracted from peripheral blood with the QIAamp^®^ DNA Blood Maxi Kit (Qiagen) according to the manufacturer’s protocol. Fragment length analysis of *SNCA* Rep1 and length determination was performed as described previously [15]. Patients with the shorter genotype (both alleles shorter than 263 base pairs (bp)) were grouped into ‘short’ and patients with the longer genotype (one or more alleles 263 bp or longer) were grouped into ‘long’. Apolipoprotein (*APOE*) genotype was assigned as described previously [19] and adjusted for in subsequent analyses due to its association with cognitive impairment in PD [20].

### Statistical analysis

Patient demographics and clinical characteristics were compared between short and long allele carriers using two-sample *t*-test or Mann-Whitney *U* test (based on normality) for continuous variables reported as mean with standard deviation or median with quartiles as appropriate; and chi-squared test or Fisher’s exact test (where appropriate) for categorical variables reported as frequency and percent. Analysis of covariance was conducted, adjusting for age, gender, disease duration, baseline MoCA score, H&Y stage and *APOE4* status, to determine the association between Rep1 polymorphism and clinical outcomes at baseline. Associations between Rep1 length and progression of non-motor symptoms over 4 years was examined for global cognition (MMSE, MoCA), non-motor outcomes (10 tests), and 9 subdomains in NMSS. These were analyzed by testing the interaction term of Year x Rep1 status in linear mixed models (LMM), while single terms of Year and Rep1 status were included in the LMM investigating the mean change of the outcomes over time, adjusted for potential confounders. Variance components covariance structure was used for the random intercept-only model and restricted maximum likelihood approach as the estimation method. Beta coefficients (regression slopes) and least-square means (mean over time) and their corresponding 95% confidence intervals (CI) were reported. Statistical significance was set at *p* < 0.05, with Bonferroni correction for multiple testing. All statistics were performed using SPSS version 25 (IBM).

### Data availability

Anonymised data that support the findings of this study are available from the corresponding author upon reasonable request.

## Supplementary Materials

Supplementary Figure 1
