# Supplementary material for: Carriers of Parkinson’s disease-linked SNCA Rep1 variant have greater non-motor decline: a 4 year follow up study
Source: Aging (Albany NY). 2025 Feb 3;17(2):357–64. doi: 10.18632/aging.206196 (PMC11892927; doi:10.18632/aging.206196)
Supplement: Supplementary Figure 1 [file aging-17-206196-s001.pdf]

SUPPLEMENTARY FIGURE

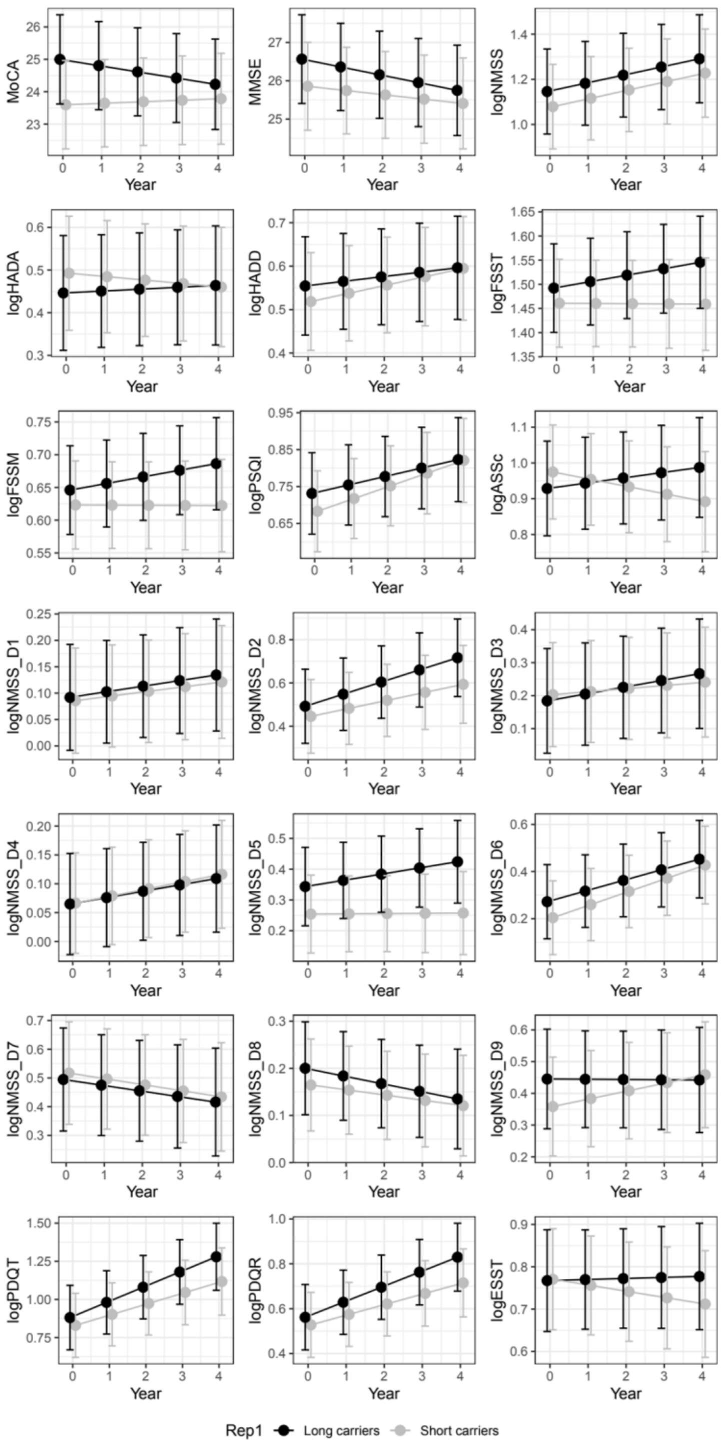

Supplementary Figure 1. Associations between SNCA Rep1 allele length and non-motor symptoms (including global cognition) in early PD patients over time. Results are generated using linear mixed model analysis, controlling for age, gender, disease duration, *APOE4* status, baseline H&Y status (for MMSE and MoCA scores) and baseline MoCA scores (for all other non-motor functions).
